# An Energy-Aware Task Scheduling for Quality-of-Service Assurance in Constellations of Nanosatellites

**DOI:** 10.3390/s22103715

**Published:** 2022-05-13

**Authors:** Laio Oriel Seman, Brenda F. Ribeiro, Cezar A. Rigo, Edemar Morsch Filho, Eduardo Camponogara, Rodrigo Leonardi, Eduardo A. Bezerra

**Affiliations:** 1 Graduate Program in Applied Computer Science, University of Vale do Itajaí (UNIVALI), Itajaí 88302-901, SC, Brazil; 2 Department of Automation and Systems Engineering, Federal University of Santa Catarina (UFSC), Florianópolis 88040-900, SC, Brazil; eduardo.camponogara@ufsc.br; 3 Department of Electrical Engineering, Federal University of Santa Catarina (UFSC), Florianópolis 88040-900, SC, Brazil; brenda.r@posgrad.ufsc.br (B.F.R.); cezar.rigo@spacelab.ufsc.br (C.A.R.); eduardo.bezerra@ufsc.br (E.A.B.); 4 Graduate Program in Aerospace Engineering, Federal University of Maranhão (UFMA), São Luís 65080-805, MA, Brazil; edemar.filho@ufma.br; 5 Brazilian Space Agency (AEB), Brasilia 70610-200, DF, Brazil; rodrigo.leonardi@aeb.gov.br

**Keywords:** CubeSat, mission planning, energy management, optimization

## Abstract

When managing a constellation of nanosatellites, one may leverage this structure to improve the mission’s quality-of-service (QoS) by optimally distributing the tasks during an orbit. In this sense, this research proposes an offline energy-aware task scheduling problem formulation regarding the specifics of constellations, by considering whether the tasks are individual, collective, or stimulated to be redundant. By providing such an optimization framework, the idea of estimating an offline task schedule can serve as a baseline for the constellation design phase. For example, given a particular orbit, from the simulation of an irradiance model, the engineer can estimate how the mission value is affected by the inclusion or exclusion of individuals objects. The proposed model, given in the form of a multi-objective mixed-integer linear programming model, is illustrated in this work for several illustrative scenarios considering different sets of tasks and constellations. We also perform an analysis of the Pareto-optimal frontier of the problem, identifying the feasible trade-off points between constellation and individual tasks. This information can be useful to the decision-maker (mission operator) when planning the behavior in orbit.

## 1. Introduction

Since the last decade, there has been an expressive growth in the number of small satellites launched into space. Proportionally, the very small satellites category represents three in four spacecraft launched between 2011 and 2020. Such a tendency can be attributed to the miniaturization and standardization trends that affect the electronic sector, resulting in reduced development and manufacturing costs. In the context of space missions, the standardization also strongly impacts the launch costs, which, in some cases, can be greater than the satellite cost itself [1].

A notable nanosatellite standard is the CubeSat, developed in 1999 at California Polytechnic State University. A 1U CubeSat consists of a 10 cm edge cube, weighing no more than 2 kg [2]. The model has become more popular with each year due to its construction based on components off-the-shelf (COTS) and its release to space using a standardized deployer, which significantly reduces the overall associated costs [3]. The new space era is characterized by greater accessibility, whether in an academic context or for the industry in general. This scenario favors applications aimed at research, such as FloripaSat-I [4], the CubeSat developed and built by Brazilian students at the Federal University of Santa Catarina (UFSC), and the Catarina constellation, a set of nanosatellites currently being developed by public and private entities from the State of Santa Catarina, in the southern region of Brazil, which aims to serve the agricultural and national civil defense sectors as well as foster the local space industry [5].

Constellation missions are a trending application of the CubeSat standard and, thus far, comprise half of the CubeSats ever released into orbit [6]. They are perceived as a powerful way to explore the space environment due to their ability to cover large areas and capture data from multiple points simultaneously [7]. In contrast to the scientific and commercial capabilities of these affordable small satellites, challenges regarding their reduced dimensions and weight arise. Owing to their slighter size and, consequently, smaller surface area, CubeSats have a lower capacity for harvesting and storing energy, which makes the constantly evolving electrical power system (EPS), a major subsystem, achieve better efficiency within certain weight, size, and cost limits. Such limitations, along with the fact that small satellite missions typically last less time than larger satellite missions, highlight mission planning as a cheap solution capable of improving the overall mission value by optimizing the use of available power resources [8]. This optimal planning can be achieved by scheduling the tasks to be performed through the combination of a representative mathematical model and an optimization algorithm, addressed as the task scheduling problem.

Although the single-satellite energy-constrained task scheduling problem has been addressed in the existing literature [9], this work approaches the constellation specifics in a novel way, since one may leverage this structure to improve the mission’s QoS by optimally distributing the tasks among satellites during an orbit.

By considering, in addition to power limitations, different types of tasks: collective, individual, and with stimulated redundancy; this research proposes an offline energy-aware task scheduling problem formulation regarding the specifics of constellations. In this sense, a multi-objective optimization problem is proposed through an exact mixed-integer linear programming (MILP) solver. Furthermore, we perform an analysis of the Pareto-optimal frontier of the problem, identifying the feasible trade-off points between constellation and individual tasks. This information can be useful to the decision-maker (mission operator) when planning an orbit behavior.

By providing such an optimization framework, the idea of estimating an offline task schedule can serve as a basis for the constellation design phase. For example, given a particular orbit, from the simulation of an irradiance model, the engineer can evaluate in an estimated way how the mission value is affected by the inclusion or exclusion of individuals objects. Thus, the engineer can, for example, choose to include or remove a payload, prohibit the execution of tasks simultaneously, enable the redundancy of tasks, and overall better understand the bounds of the constellation.

The main contributions of this work are:

- A novel methodology for optimal task scheduling for constellation of nanosatellites, through a model that extends on that proposed by [9];
- Concept development for aiding engineers in the constellation design phase;
- An analysis of the trade-off between individual and collective satellite tasks.

This paper is structured into sections: Section 2 reviews related research regarding the application of optimization algorithms to nanosatellites; Section 3 describes the developed optimization model, detailing its inputs, the multi-objective function to be optimized, and the constraints; Section 4 discusses the results obtained from illustrative scenarios; finally, Section 5 deliberates over the final considerations.

## 2. Related Works

This section addresses different formulations and applications of the scheduling problem on multi-satellite mission optimization. Here, the following aspects of interest are observed: the main purpose/application; whether the formulation has power constraints and/or deals with multiple objectives; how the multi-satellite dynamic is approached; and what method is used for solving the scheduling problem.

Earth observation (EO) missions represent a typical application field of task scheduling. Cui and Zhang [10] propose a dynamic scheduling model based on mission priority for emergency earth observation with multiple satellites, which allows the admission of newly arrived tasks. This multi-objective application is energy constrained and targets: the maximization of the sum of mission priorities, maximization of total mission revenues, and minimization of waiting time for missions requiring urgent execution. The solution is obtained through a hybrid genetic tabu search algorithm; therefore, it is not precise. Moreover, EO with energy constraints and multiple objectives are addressed by Lei et al. [11]. In their work, similar to [10], there is only one solution—not a set, i.e., the objectives are not mutually exclusive—and it is also not obtained through an exact algorithm; instead, the authors propose a heuristic hierarchical scheduling method based on ant colony. An exact MILP formulation is presented by Xiaoyu et al. [12] for EO applications but with no energy constraints or multiple-objective considerations.

Data transfer is another commonly addressed issue. Xiaohua et al. [13] suggest an iterative graph-based heuristic for maximizing data downloading from Ground station within its contact window with the multi-satellite system. Only download and offload times are considered resources; therefore, the model is not energy constrained. Data transfer and battery usage are both optimized in the work of [14], but the presented approach is not easily generalizable since it is based on a specific CubeSat constellation from the Danish mission called Ulloriaq.

Monmousseau [15] created a mixed-integer scheduling model within a set of possible events for a constellation of imaging satellites that did not use a heuristic solving method. His linear model enabled a better understanding of the performance of the simulated annealing solver and could also be adapted to different real-world scheduling problems. Each satellite could have different events with diverse utilities and costs in the model. The results showed the possibility to model the scheduling problem for a constellation of up to 30 satellites as a mixed-integer linear model, and the model could be improved to the point of being more time-efficient than a simulated annealing process.

The work of Cho et al. [16] proposed a mixed-integer linear program formulation for the job scheduling of a constellation and investigated the applicability/scalability of a standard MILP solver that produces the optimal solution. The goal of the satellite constellation job scheduling was to allocate each job for satellites and determine the job starting times to maximize overall mission performance. The constraints included visibility time windows, transition time between consecutive jobs, maximum attitude angle, energy capacity, and memory capacity. The formulation relaxed some of the constraints, which would not impact real instances, but also included precedence conditions between jobs and job-agent compatibility constraints. An off-the-shelf MILP solver was used to obtain the optimal solution for the scheduling formulation and numerical experiments indicated that the optimal solution can be obtained in a tractable manner up to the problem size with three satellites and hundreds of jobs.

A formulation aiming to maximize the overall mission performance metric of low-Earth-orbit satellite constellations is proposed by Cho et al. [17]. Their model considers both data transfer and earth observation; in fact, the authors divide the tasks into two types: observation tasks and download tasks. The exact solution to the presented scheduling problem is obtained through a two-step binary linear programming method combined with an off-the-shelf MILP solver. However, as in [10,11], the model does not allow a Pareto-optimality analysis, i.e., the objectives are combined into one objective-function with a single optimal solution.

The Pareto optimality is addressed by the work of Rocco, Souza, and Prado [18], in which orbital maintenance of symmetrical constellations is considered, along with minimal fuel consumption. For solving the problem, the authors propose a new method called smallest loss criterion, based on Pareto optimization. The method proposes that the optimal solution in a multi-objective—with conflicting objectives-problem—should be the one with the smaller loss for all the objectives, therefore, not prioritizing or neglecting any specific objective. The formulation does not concern energy availability since the resource necessary for performing the maneuvers is fuel.

Rigo et al. [9] proffer a concise model of which generality and energy constraints—irradiance and battery models considered—are distinguished features. The research presented in this paper extends on the formulation from [9] by covering constellation specificities, including dynamic priorities, and analyzing the problem in a multi-objective Pareto-optimality manner, therefore, delivering a general-purpose model applicable to CubeSat constellations planning, such as the Catarina constellation [5].

## 3. Problem Statement

Our paper presents details regarding a new mathematical formulation for QoS of nanosatellites, which was formulated to account for the behavior of constellations. The proposed formulation extends on the limitations presented in the previous work of Rigo et al. [9], whose constraints—despite accounting for the energy-aware behavior of the object—only considered a single nanosatellite. In this regard, it is possible to explore the Pareto-frontier achieved from a trade-off between individual and collective tasks (i.e., constellation tasks). In other words, the model aims at achieving broad applicability, in order to serve as a tool for planning multi-satellite space missions. The main additions to the original model are listed below:

- Model expanded for multiple satellites;
- Separation between individual and collective (constellation) tasks;
- Possibility of redundancy;
- Analysis of the Pareto-frontier for the trade-off between individual and constellation tasks.

Table 1 details the notation of the sets and variables. Model inputs are defined in Table 2. The following parts of this section discuss the objective functions, model constraints, and an auxiliary irradiation model we adopted for simulating energy availability in CubeSats.

### 3.1. Irradiation

The available power from the solar panels in Equation (1) is obtained through the irradiance model developed in [19]. The model provides the flux of irradiation ${G\prime \prime }_{t}$ (W/m^2^) obtained from a combination of orbital parameters and spin of the CubeSat (also known as attitude), which is here combined with the solar panel’s area ($A_{w}$) of each side (*w*) and the efficiency (η) of the cells. The standard CubeSat has six sides, and for this reason, the summation is from one to six.

(1) $$r_{t}=\sum_{w=1}^{6}\eta A_{w}G_{t}^{\prime \prime }$$

### 3.2. Model

The subset of tasks (or jobs) j∈C refers to the group of *constellation tasks*, i.e., the ones assigned to all the satellites which any can execute. The tasks that do not belong to subset C are called *individual tasks* ( I) and are assigned to a single satellite. An optimal allocation of the available energy resources must distribute them between both described task groups—constellation and individual. However, since the resources are limited, a group of tasks can not be favored—with more allocated resources—without deteriorating the other group. Therefore, there are multiple optimal solutions to a problem in this form. By splitting the cost function *F* defined in Equation (2) into two terms—*A* and *B*, respectively, corresponding to constellation tasks and individual tasks—a certain degree of flexibility is achieved. This allows the application of methodologies such as the ϵ-constrained method presented in [20], which is capable of identifying the Pareto-frontier of the problem. Maximizing *F* means optimizing energy usage by scheduling tasks according to their priorities $u_{s,j,t}$, therefore, improving the mission value and the quality of service (QoS).

(2) $$\begin{array}{c} F:\;\;\max_{x_{s,j,t}}\;\;\underset{A}{\underset{︸}{\sum_{(s,j,t)\in C}u_{s,j,t}x_{s,j,t}}}+\underset{B}{\underset{︸}{\sum_{(s,j,t)\in I}u_{s,j,t}x_{s,j,t}}}+\underset{C}{\underset{︸}{\sum_{(s,s^{\prime },j,t)\in R\subset C}\beta_{s,s^{\prime },j,t}}} \end{array}$$

Notice that while the *C* term is presented at the cost function, it will not be considered in the ϵ-constrained methodology, being in charge of directing the redundancy of the tasks, which will be discussed later.

Constraints (3a)–(15a) integrate the model to guarantee that the scheduled tasks: respect the power limitations, (optionally) provide battery lifetime preservation and consider task properties, i.e.,: dynamic priority; minimum and maximum (min. and max.) startups in a single satellite (or subsystem); min. and max. startups for the constellation as a whole (general startups); min. and max. number of subsystems to perform a task simultaneously; (optional) encouraged simultaneity (redundancy); execution window; min. and max. execution time; min. and max. period between task runs. The constraints are categorized and presented in the following subsections.

#### 3.2.1. Auxiliary Variables

**Figure 1:**
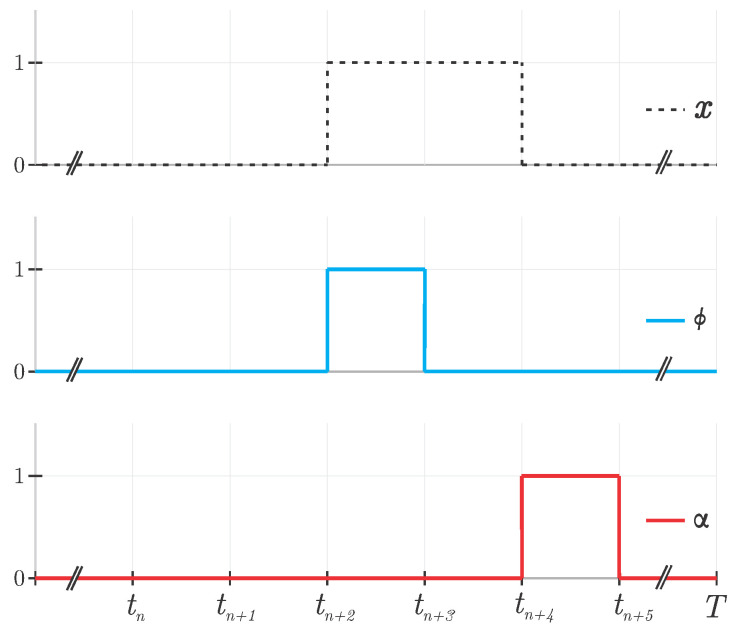
Behavior of auxiliary variables ϕ and α in relation to a given job *x*.

Two auxiliary binary variables, ϕ and α, identify, respectively, whenever a job starts and when it ends. The first of them, ϕ is given by constraints (3a)–(3d), and represents the moment in time when the task starts; the second of them, α, is given by constraint (4a)–(4c), and represents the first moment in time that the job is not operating, i.e., has already finished. Figure 1 illustrates the expected behavior of the constraints.

(3a) $$\begin{array}{cc} \; & \phi_{s,j,t}\geq x_{s,j,t},\;\forall j,\forall t=1,\forall s \end{array}$$

(3b) $$\begin{array}{cc} \; & \phi_{s,j,t}\geq x_{s,j,t}-x_{s,j,(t-1)},\;\forall j,\forall t>1,\forall s \end{array}$$

(3c) $$\begin{array}{cc} \; & \phi_{s,j,t}\leq x_{s,j,t},\;\forall j,\forall t,\forall s \end{array}$$

(3d) $$\begin{array}{cc} \; & \phi_{s,j,t}\leq 2-x_{s,j,t}-x_{s,j,(t-1)},\;\forall j,\forall t>1,\forall s \end{array}$$

(4a) $$\begin{array}{cc} \; & \alpha_{s,j,t}\geq x_{s,j,(t-1)}-x_{s,j,t},\forall j,\forall t>1,\forall s \end{array}$$

(4b) $$\begin{array}{cc} \; & \alpha_{s,j,t}\leq x_{s,j,(t-1)},\forall j,\forall t>1,\forall s \end{array}$$

(4c) $$\begin{array}{cc} \; & \alpha_{s,j,t}\leq x_{s,j,(t-1)}-x_{s,j,t}+\phi_{s,j,t},\forall j,\forall t>1,\forall s \end{array}$$

notice that $\alpha_{s,j,1}=0$ since the task cannot end at the first time step.

#### 3.2.2. Running Time and Startups

An execution window, associated with a job *j* and a subsystem *s*, is assured through constraints (5a) and (5b), which prevent task startups outside the time window $\left[w_{s,j}^{\min},w_{s,j}^{\max}\right]$ by preventing values of $x_{s,j,t}$ other than zero.

(5a) $$\begin{array}{cc} \sum_{t=1}^{w_{s,j}^{\min}}x_{s,j,t} & =0,\;\forall j,\forall s \end{array}$$

(5b) $$\begin{array}{cc} \sum_{t=w_{s,j}^{\max}+1}^{T}x_{s,j,t} & =0,\;\forall j,\forall s \end{array}$$

With the aid of the previously introduced variable $\phi_{s,j,t}$, a minimum and maximum time between startups of a given task are defined, respectively, by constraints (6a) and (6b). Constraint (6a) ensures that a task is not invoked more than once in any time window of size $p^{\min}$, and constraint (6b) ensures the task is executed at least once in a given period of size $p^{\max}$.

(6a) $$\begin{array}{cc} \; & \sum_{l=t}^{t+p_{s,j,t}^{\min}-1}\phi_{s,j,l}\leq 1,\forall t\in \left\{1,...,T-p_{s,j,t}^{\min}+1\right\},\forall j,\forall s \end{array}$$

(6b) $$\begin{array}{cc} \; & \sum_{l=t}^{t+p_{s,j,t}^{\max}-1}\phi_{s,j,l}\geq 1,\forall t\in \left\{1,...,T-p_{s,j,t}^{\max}+1\right\},\forall j,\forall s \end{array}$$

Constraints (7a)–(7c) guarantee that, once a task has started, it is completed successfully, contemplating its minimum and maximum execution time. Constraint (7a) ensures that once a task is started, it respecst a minimum time of $t^{\min}$, which is only enforced when a task begins running as given in the multiplication by $\phi_{s,j,t}$ on the right side of the equation. Constraint (7b) ensures that a task runs no more than $t^{\max}$ units of time. Finally, constraint (7c) is used to enforce the desired behavior at the end of the period, i.e., if the task starts at moment equal or higher than $T-t^{\min}$, it should remain active until the end of the orbit.

(7a) $$\begin{array}{cc} \; & \sum_{l=t}^{t+t_{s,j,t}^{\min}-1}x_{s,j,l}\geq t_{s,j,t}^{\min}\phi_{s,j,t},\forall t\in \left\{1,...,T-t_{s,j,t}^{\min}+1\right\},\forall j,\forall s \end{array}$$

(7b) $$\begin{array}{cc} \; & \sum_{l=t}^{t+t_{s,j,t}^{\max}}x_{s,j,l}\leq t_{s,j,t}^{\max},\;\forall t\in \left\{1,...,T-t_{s,j,t}^{\max}\right\},\forall j,\forall s \end{array}$$

(7c) $$\begin{array}{cc} \; & \sum_{l=t}^{T}x_{s,j,l}\geq \left(T-t+1\right)\phi_{s,j,t},\forall t\in \left\{T-t_{s,j,t}^{\min}+2,...,T\right\},\forall j,\forall s \end{array}$$

The minimum and maximum number of startups within an orbit are defined for each task, respectively, by (8a) and (8b).

(8a) $$\begin{array}{cc} \; & \sum_{t=1}^{T}\phi_{s,j,t}\geq y_{s,j,t}^{\min},\;\forall j,\forall s \end{array}$$

(8b) $$\begin{array}{cc} \; & \sum_{t=1}^{T}\phi_{s,j,t}\leq y_{s,j,t}^{\max},\;\forall j,\forall s \end{array}$$

#### 3.2.3. Battery

The battery is considered in the present model through the adaptation introduced by Rigo et al. in [9], in which they propose an MILP formulation for the battery based on the Generalized Disjunctive Program (GDP) of Grossmann and Lee [21]. According to this representation, the state of charge (SoC) of the battery is defined by constraints (9a)–(9g).

(9a) $$\begin{array}{cc} \; & {\mathrm{SoC}}_{s,t+1}\geq {\mathrm{SoC}}_{s,t}+\frac{i_{s,t}\times e_{c}}{60\;Q}-M\times \left(1-b_{s,t}\right),\;\forall t \end{array}$$

(9b) $$\begin{array}{cc} \; & {\mathrm{SoC}}_{s,t+1}\leq {\mathrm{SoC}}_{s,t}+\frac{i_{s,t}\times e_{c}}{60\;Q}+M\times \left(1-b_{s,t}\right),\;\forall t \end{array}$$

(9c) $$\begin{array}{cc} \; & {\mathrm{SoC}}_{s,t+1}\geq {\mathrm{SoC}}_{s,t}+\frac{i_{s,t}\times e_{d}}{60\;Q}-M\times b_{s,t},\;\forall t \end{array}$$

(9d) $$\begin{array}{cc} \; & {\mathrm{SoC}}_{s,t+1}\leq {\mathrm{SoC}}_{s,t}+\frac{i_{s,t}\times e_{d}}{60\;Q}+M\times b_{s,t},\;\forall t \end{array}$$

(9e) $$\begin{array}{cc} \; & i_{s,t}\leq M\times b_{s,t},\;\forall t \end{array}$$

(9f) $$\begin{array}{cc} \; & i_{s,t}\geq -M\times \left(1-b_{s,t}\right),\;\forall t \end{array}$$

(9g) $$\begin{array}{cc} \; & b_{s,t}\in \left\{0,1\right\},\;\forall t \end{array}$$

where $i_{s,t}$ is the total current of a satellite *s* in a given time step *t*. Notice that this current can be either positive or negative, indicating the direction of the energy balance $b_{s,t}$, which is then used to define the efficiency to be used in the SoC approximation, in other words, the efficiency parameter $e_{c}$ is considered if the energy balance is positive, while the efficiency parameter $e_{d}$ is used if the energy balance is negative (i.e., the battery is discharging).

The overall SoC variation is limited by constraint (10), which implies restraining battery usage.

(10a) $$\begin{array}{c} {\mathrm{SoC}}_{s,t}\leq 1,\forall s,\forall t \end{array}$$

(10b) $$\begin{array}{c} {\mathrm{SoC}}_{s,t}\geq \rho ,\;\forall s,\forall t \end{array}$$

Finally, as stated in (11), for each instant of time, the sum of the resources demanded by the active tasks is restricted to the sum of the maximum available power from the solar panels and a portion of the energy resources available in the battery.

(11a) $$\begin{array}{cc} \; & \sum_{s=1}^{S}\sum_{j=1}^{J}q_{s,j}x_{s,j,t}\leq r_{t}+\gamma \;V_{b}\;\left(1-\lambda_{s,t}\right),\;\forall t \end{array}$$

(11b) $$\begin{array}{cc} \; & 0\leq \lambda_{s,t}\leq 1,\;\forall t \end{array}$$

where λ is a decision variable regarding the amount of resources to be used from the battery, considering a fixed battery voltage $V_{b}$ and a fixed battery draining current γ, in other words, a total of $\gamma V_{b}$ units of power are used.

#### 3.2.4. Constellation

As the constellation tasks can be performed by any satellite, constraint (12) restricts the minimum and maximum number of subsystems to be conducting a job *j* at an instant *t*. Such constraints establish a minimum and maximum simultaneity factor ψ.

(12a) $$\begin{array}{cc} \; & \sum_{s=1}^{S}x_{s,j,t}\geq \psi_{j}^{\min},\;\forall t,\forall j \end{array}$$

(12b) $$\begin{array}{cc} \; & \sum_{s=1}^{S}x_{s,j,t}\leq \psi_{j}^{\max},\;\forall t,\forall j \end{array}$$

Minimum ($g_{j}^{\min}$) and maximum ($g_{j}^{\max}$) general startups are defined for constellation tasks, as stated by constraints (13a) and (13b). Notice that these constraints work the same way as the previously presented (8a) and (8b), with the included consideration of all the constellation satellites.

(13a) $$\begin{array}{cc} \; & \sum_{s=1}^{S}\sum_{t=1}^{T}\phi_{s,j,t}\geq g_{j}^{\min},\;\forall j \end{array}$$

(13b) $$\begin{array}{cc} \; & \sum_{s=1}^{S}\sum_{t=1}^{T}\phi_{s,j,t}\leq g_{j}^{\max},\;\forall j \end{array}$$

A given subset of tasks, i.e., Ξ⊂C, can leverage from network behavior, i.e., run synchronously within the entire constellation (all satellites, |S|) at the same time. Such behavior can be enforced by a simultaneous start and ending, as in constraints (14a) and (14b).

(14a) $$\begin{array}{cc} \; & \sum_{s^{\prime }\in S}\phi_{s^{\prime },j,t}\geq \left|S\right|\phi_{s,j,t},\forall t,\forall j,\forall s\in \Xi \end{array}$$

(14b) $$\begin{array}{cc} \; & \sum_{s^{\prime }\in S}\alpha_{s^{\prime },j,t}\geq \left|S\right|\alpha_{s,j,t},\forall t,\forall j,\forall s\in \Xi \end{array}$$

In order to achieve a system of redundancy in the execution of tasks, we introduce the binary variable β, which is equal to 1 when two tasks are running simultaneously in two different satellites, and equal to 0 when they are not (which is given by means of a big-M formulation). This identification, which is used to enforced mission reliability, is given by:

(15a) $$\begin{array}{cc} \; & \phi_{s,j,t}+\phi_{s^{\prime },j,t}\geq 2-M\left(1-\beta_{s,s^{\prime },j,t}\right),\forall t,\forall j,\forall s\in R,\forall s^{\prime }\in R \end{array}$$

notice that the redundancy is enforced only for a specified subset R⊂C.

### 3.3. Solving the Proposed Model

The optimal schedule of a set of constellation tasks, including both individual and collective tasks, is achieved by solving the problem formally defined below:

(16) $$\begin{array}{cc} F:\;\max_{x_{s,j,t}}\;\; & \left(2\right)\\ s.t.\; & \;(3a)–(15a) \end{array}$$

The resulting problem is an MILP (Mixed-Integer Linear Programming) problem, which has its general form given by:

(17a) $$\begin{array}{cc} \min\; & c^{T}x+h^{T}y \end{array}$$

(17b) $$\begin{array}{cc} s.t.\; & H_{k}x+G_{k}y\leq b_{k},\;\forall k\in \left\{1,\ldots ,m\right\} \end{array}$$

(17c) $$\begin{array}{cc} \; & x\in Z^{n},y\in R^{p} \end{array}$$

where the coefficients of the vectors c and h are easily obtained from (16), H and G are matrices and b is a vector which are all obtained from the aforementioned *m* constraints.

Typically, MILP problems can be solved to optimality or near optimality (within a dual gap) by exact algorithms such as branch-and-bound [22] or approximated using heuristics. In general, MILP is an NP-Hard problem in the sense that any NP-Complete problem (e.g., the classic satisfiability problem) can be reduced to it in polynomial time. Any polynomial time algorithm for MILP would imply that P = NP [A], in other words, all decision problems would be solved in polynomial time.

**Figure 2:**
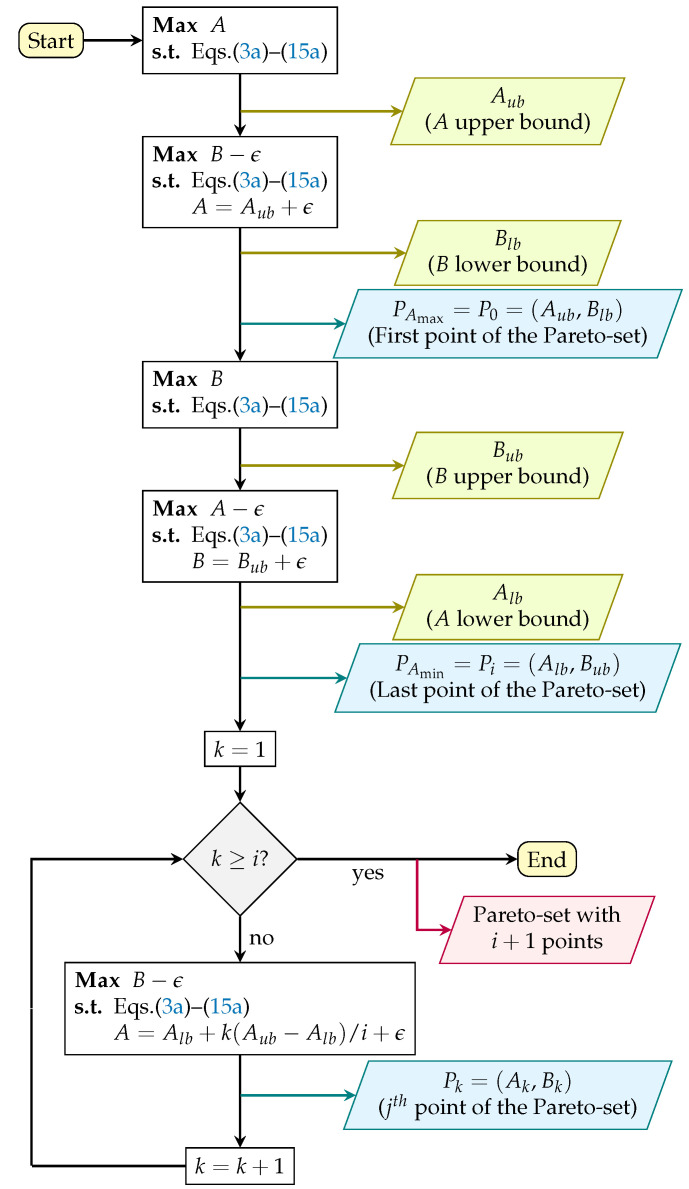
Representative flowchart of the ϵ-constrained solution procedure.

In our work, instead of solving the problem in a straightforward manner defined by Equation (17), we employ the ϵ-constrained methodology proposed by Mavrotas [20], which is illustrated in Figure 2. The methodology, employed for cost function (2), with terms A and B, is used to find the Pareto-optimal frontier of the proposed problem. From the perspective of the constellation operator, it is useful to give information about the trade-off that can be achieved by defining the priority for constellation and individual satellite tasks.

The ϵ-constrained method is known to provide efficient solutions for multi-objective mixed-integer problems. It consists of solving the problem multiple times along with a slack variable ϵ, which allows for the fixing of A or B values without rendering the problem infeasible. First, we maximize the constellation tasks (objective function term A), which provides an upper bound to the set of constellation tasks C ($A_{ub}$). Then, we solve problem (17), now maximizing the individual tasks (objective function term B), given that the constellation tasks (A) QoS remains near the previously solved upper bound (within a given threshold); this step gives the lower bound for the individual tasks ($B_{lb}$). This step is then repeated, but now first maximizing B, then maximizing A, which will give the upper bound for the individual tasks ($B_{ub}$), and the lower bound of the constellation tasks ($A_{lb}$). These bounds are then considered in a next step as being the maximum and minimum range that both terms (A and B) can reach inside the Pareto-optimal frontier.

In the next step, also illustrated in Figure 2, an iteration, guided by index *j*, produces a Pareto-frontier with resolution of i+1 points. The iterative process (over *k*) is responsible for calculating the Pareto points within the previously defined range $\left[A_{lb},A_{ub}\right]$, which relies on maximizing B in problem (17), once again ensuring that A remains within a desirable threshold, in an intermediate value between its maximum and minimum value:

(18) $$\begin{array}{c} A=A_{lb}+k\left(A_{ub}-A_{lb}\right)/i+\epsilon , \end{array}$$

whereby the obtained results are saved and used later to plot the Pareto-frontier.

## 4. Results

To demonstrate the effectiveness of our methodology in planning a nanosatellite constellation, this section brings about analyses of the features introduced to the original MILP and the corresponding results. After presenting the orbital parameters used for power harvesting prediction, we first report results regarding a simple example, consisting of a constellation with two satellites, to illustrate the consistency of the scheduling. Then an analysis of objective function values and solution time is carried out for progressively larger constellation sizes to assess the QoS impact of adding more satellites to a constellation. Pareto-optimal results for constellations with two and three satellites are presented to provide even more information to the decision-maker. Finally, an analysis of the impact of adding the redundancy term in the multi-objective function is presented.

All methodologies were implemented in the Julia programming language using the JuMP library [23] and solved for a GAP ≤ 1% using the Gurobi solver in a server with an Intel(R) Xeon(R) CPU E5-2630 v4 @ 2.20GHz processor (20 cores, 40 threads), 64 GB of RAM, and Ubuntu 20.04.2 LTS 64 bits. Supplementary material is available.

### 4.1. Energy Harvesting Calculation

The methodology presented by [19] was used for power input calculation in each satellite considering orbital parameters from the CubeSat FloripaSat-I [4], as shown in Table 3. Finally, the spacecraft’s attitude adopted here is the NADIR where one specific face of the satellite is always directed towards the Earth’s surface.

This is a nearly-circular orbit with 623 km of altitude, which results in a period of 96 min, whose fraction is spent under the Earth’s shadow where there is no power input. Each CubeSat of the constellation is in this same orbit, but equally spaced from each other, e.g., for the constellation with two spacecrafts the angular displacement (true anomaly) is 180${}^{\circ }$, while for the constellation with three satellites the displacement is 120${}^{\circ }$.

With these parameters the power input vector was obtained and multiplied by an Electrical Power System efficiency of 0.85, resulting in the power available for the tasks in each satellite—input for the MILP formulation.

### 4.2. Initial Validation

An example was created with randomly generated task requirements for a constellation with two 3U nanossatelites, as presented in Table 4, and battery parameters as presented in Table 5.

A total of 15 unique tasks are considered in this hypothetical constellation. Five of them are constellation tasks, meaning that they can run on either or both satellites, and five unique and individual tasks can be executed only by the owner satellite.

**Figure 3:**
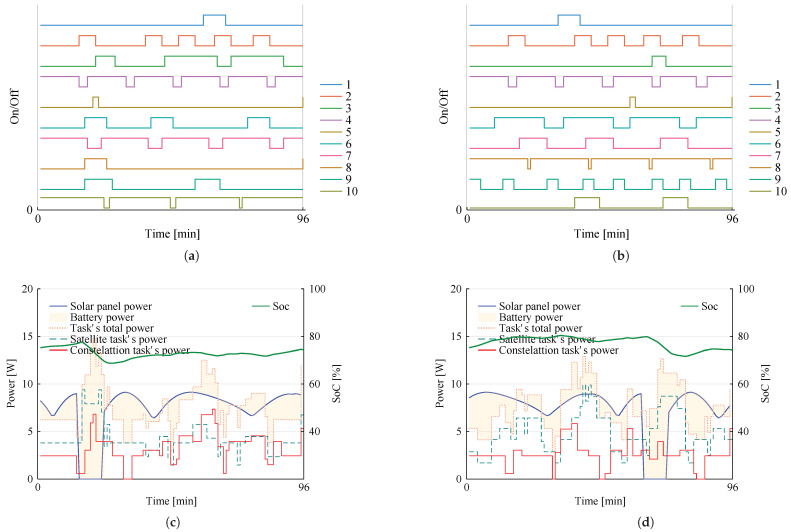
Mission planning for a two-satellite constellation. (**a**) Scheduling for satellite 1. (**b**) Scheduling for satellite 2. (**c**) Energy analysis for satellite 1. (**d**) Energy analysis for satellite 2.

The mission plan obtained for an orbit in this constellation is presented by Figure 3, in which Figure 3a,b show the scheduling for each task in each satellite—satellite 1 and satellite 2, respectively. Notice that tasks are labeled from 1 to 10, the first five (1 to 5) being constellation tasks, and the remaining (6 to 10) individual. Furthermore, task 2 is required to be synchronous.

From the results shown in Figure 3, we can observe that the task schedule is consistent with the requirements of each task. Task 2, for instance, has the highest priority among the constellation tasks, being accordingly invoked five times in each satellite which is the maximum number of activations allowed ($y_{j}^{\max}$). Moreover, we can verify that task 2 starts and finishes execution at exactly the same time in both satellites, as it must since this task is synchronous. In contrast, task 1 bears the lowest priority, being invoked the minimum number required, which in this case is not $y_{j}^{\min}$ (0) but the global minimum, $g_{j}^{\min}$ (2).

Figure 3c,d show the power footprint for this mission plan. The solid blue line represents power coming directly from the solar harvesting system for each orbit instant, including the eclipse period when it drops to zero. Note that the eclipse time does not occur simultaneously in both satellites, since we consider each satellite to be equidistant from the others on the orbit plane; hence, when one is in eclipse, another is not—a property which is ensured by all problem instances considered in this paper. Conversely, the solid red line represents the total energy consumption by constellation tasks, whereas the dashed teal line tracks the full energy usage of the satellite’s tasks. The dotted coral line shows the total power consumption at any given instant, along with the wheat-colored shade that indicates whether the power is being drawn from or stored in the battery, together with the consequent SoC impact represented by the solid green line.

Compared with the schedule, we can verify that this power behavior is consistent with the schedule in each satellite, directly correlated with the on/off of each task. The battery power is most intensely used in eclipse time, as expected, whereby the state-of-charge drops accordingly in this period, while not violating any of the constraints (10).

**Figure 4:**
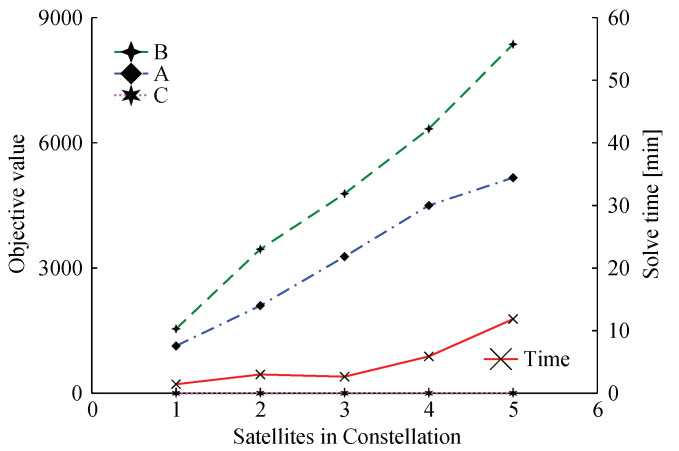
Collective (A) and individual (B) task objective values and respective solving time for five constellation sizes—from one to five satellites.

Now let us investigate how the objective functions A and B values and solve time varies when increasing the constellation size. For this, we maintained the task parameters constant presented in Table 4 for the constellation tasks and randomly created new task parameters for each of the satellites added. The task parameters were randomly generated taking as a basis the total duration or the orbit (*T*), with the min/max startups within a range of [T/45,T/15], the min/max CPU time within a range of [T/10,T/4], and the min/periods within a range of [T/4,T]. Figure 4 shows the results for constellation sizes varying from one to five satellites.

QoS capabilities for the constellation increase with the addition of more satellites, in an almost direct correlation, as shown by the blue line in Figure 4. However, this A/B trade-off is based on tasks priorities and, if the individual tasks in a given satellite have higher priorities, then constellation tasks will receive fewer resources, as is the case for a constellation of size five. Furthermore, as one adds more individual tasks for each of the new satellites, the number of unique tasks in the constellation also rises, consequently, raising the effort to find the optimal solution. This explains the solve time increase as the number of satellites increases.

### 4.3. Pareto-Optimal Analysis

**Figure 5:**
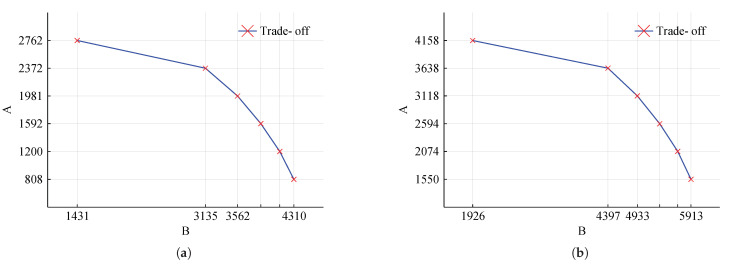
Pareto-optimal solutions for two constellations of varying sizes, considering the objective value for collective (A) and individual (B) tasks obtained withing a GAP ≤ 1%. (**a**) Constellation with two satellites. (**b**) Constellation with three satellites.

To assess the trade-off between objectives A and B more clearly, here we present the Pareto-optimal analysis for constellations of size 2 and 3. The result is shown in Figure 5.

From Figure 5a one can obtain the range of values that A and B can receive, while maintaining a Pareto-efficient solution for the constellation with two satellites. Notice that, since all points are optimal, this information only carries meaning to the decision-maker who, endowed with additional information and preferences related to the mission purpose, can select the best trade-off.

**Figure 6:**
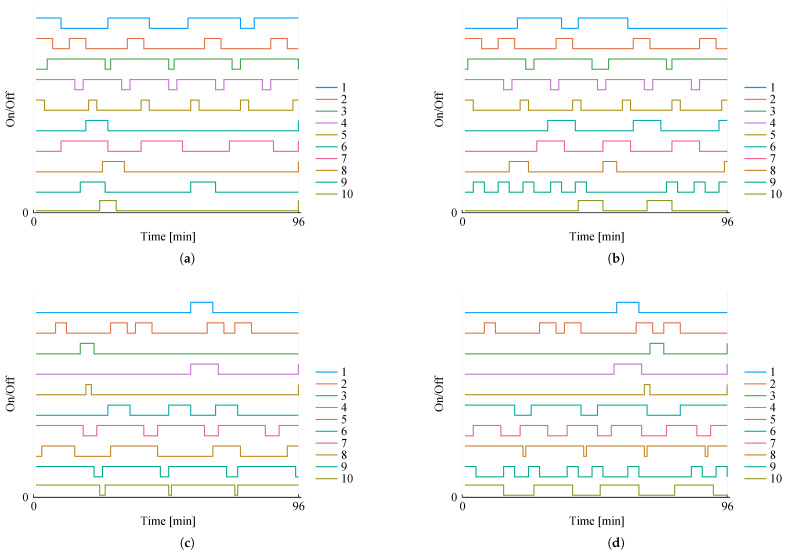
Mission planning for a two-satellite constellation considering the two Pareto-optimal extreme points: maximizing A (tasks 1 to 5) and maximizing B (tasks 6 to 10). (**a**) Scheduling for satellite 1, maximizing A. (**b**) Scheduling for satellite 2, maximizing A. (**c**) Scheduling for satellite 1, maximizing B. (**d**) Scheduling for satellite 2, maximizing B.

For illustration purposes, we show the schedule at the Pareto extremes—when maximizing for A and when maximizing for B—for the constellations with two satellites. Tasks 1 to 5 are clearly maximized, as seen in Figure 6a,b; task 1 for instance executes six times in the constellation, while it was previously invoked only two times—Figure 3. Conversely, the satellite individual tasks, 6 to 10, are maximized in Figure 6c,d.

### 4.4. Tasks Redundancy for Fault Tolerance

**Figure 7:**
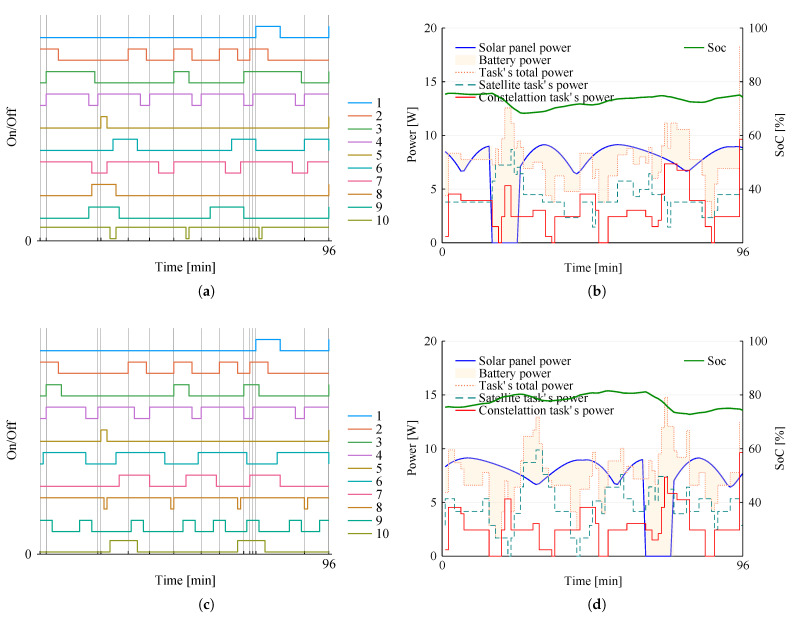
Mission planning for the two-satellite constellation, accounting for term C in the objective function. (**a**) Scheduling in satellite 1. (**b**) Energy in satellite 1. (**c**) Scheduling in satellite 2. (**d**) Energy in satellite 2.

Here we explore the effects of the objective-function term C (2) on the scheduling results. For this, we solved the problem instance consisting of constellations with two satellites, while adding term C in the objective function with a weight of 10. The resulting schedule is presented in Figure 7, along with the energy analysis.

Notice that Figure 7a,c show each instant β assumed value 1 meaning a specific constellation task started running on more than one satellite simultaneously. Comparing this result with the previous result reported in Figure 3, without C, we can see that now fifteen tasks start running simultaneously, while none were invoked before to run simultaneously. While C is an effective approach to introduce redundancy and fault tolerance in the constellation, its impact on energy dynamics is minimal, as seen in Figure 7b,d, showing a consistent power usage that adheres to constraints (10), very similar to the behavior depicted in Figure 3c,d.

**Figure 8:**
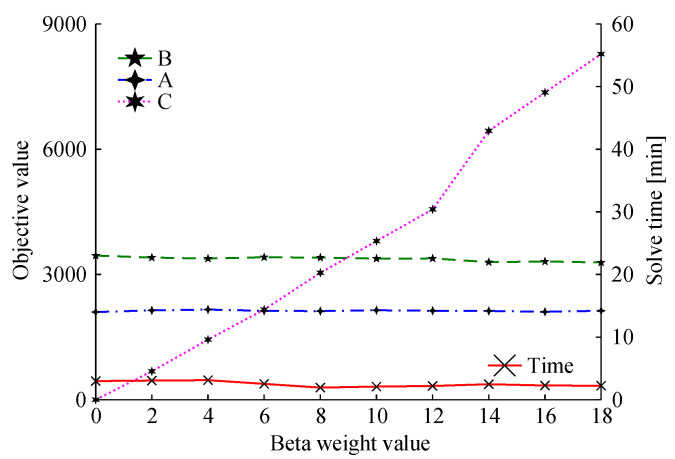
Collective (A), individual (B) and redundancy (C) objective values, and the respective solving time, for the constellation with two satellites, solved with different weight values for the objective term C.

It is relevant to assess the impact that different values of the C weight can have on the values of the objective function terms and the solving time for a given problem. For this, the problem instance of size 2 is solved using 10 different weight values, ranging from 0 to 18. The results are shown in Figure 8.

It can be observed that as the weight increases, the value of the objective-function term C also increases, but not always proportionally, which would indicate no gains in terms of redundancy—as the term C is not directly proportional to the weight, then the β variable must be increasing. If more weight is given to term C, the solving time, term A, and term B values decrease modestly, remaining practically stable. This indicates that the term C only encourages redundancy, without causing a significant loss in the values that can be extracted from the mission.

**Figure 9:**
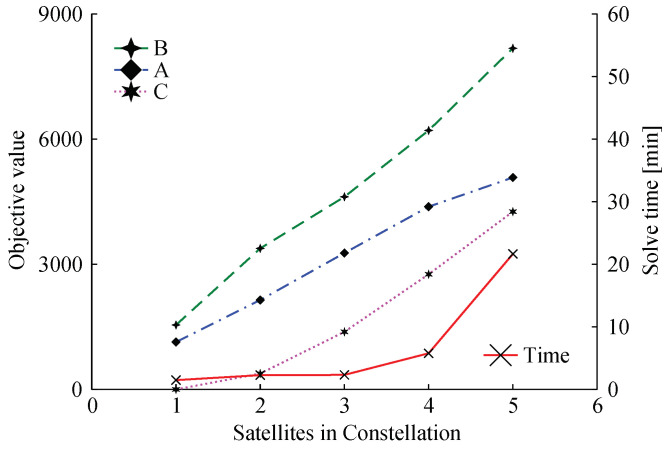
Collective (A), individual (B) and redundancy (C) objective values with the respective solving time for varying sizes of the constellation—from one to five satellites.

Finally, we study how the introduction of term C impacts on term A, term B and the solve time as the constellation size increases. The same experiment depicted in Figure 4 is repeated here, although now with C and a weight of 10. The results are depicted in Figure 9. We can conclude that A and B change only marginally, while the solution time increases considerably for size 5. Furthermore, as more satellites are admitted, more redundancy is achieved as term C increases although its weight remains constant.

## 5. Conclusions

Due to limited energy resources available in the nanosatellites, task scheduling has become an even greater challenge regarding constellations. When managing constellations, it is important to maintain the QoS of not just individual satellites, but mission objectives (tasks) as a whole. In this context, this article proposes a mathematical formulation capable of maximizing the quality of a nanosatellite mission, including consideration of the trade-off (Pareto-frontier) between individual and collective tasks. Among the functionalities of the proposed formulation, we introduced optional constraints that stimulate task redundancy to increase mission reliability.

Illustrative scenarios were simulated in order to explore the proposed formulation. The results revealed that it was possible to maintain the quality of service and the requirements associated with collective (constellation) tasks through the correct distribution of tasks among the nanosatellites. As expected, considering the same mission requirements, it can be seen that there is an increase in the quality of service as more nanosatellites are introduced into the constellation.

When the Pareto-frontier was considered, it was observed that the formulation was capable of identifying the feasible trade-off points between constellation and individual tasks. This information can be useful to the decision-maker (mission operator) when planning and managing orbit behavior.

Finally, the task redundancy was introduced in the simulated scenarios. Regarding this matter, it was possible to note that the proposed formulation initially relocated the tasks to run at the same time (redundancy), i.e., the number of tasks and executions remained the same. With the increased prioritization of redundancy, it was possible to notice a decrease in the quality of service in favor of the simultaneous execution of tasks. In this sense, the formulation once again provides the decision-maker with a trade-off in information, this time considering QoS × redundancy (realiability).

For future works, we suggest considering a decomposition for the proposed formulation, i.e., branch-and-price. Such a composition could allow the formulation to manage a larger constellation within a desirable computational time.

## Figures and Tables

**Table 1:** Sets and model variables.

Notation	Description
***General***	
*S*	the number of subsystems.
*J*	the number of tasks/jobs.
*T*	the size of T.
***Sets***	
S	Set of subsystems (satellites). S={s|s∈N,s≤S}.
J	Set of jobs (or tasks). J={j|j∈N,j≤J}.
T	Set of units of time. T={t|t∈N,t≤T}.
***Subsets***	
C	Set of constellation tasks. C⊂J.
I	Set of individual tasks. I⊆(J∖C).
Ξ	Set of simultaneous tasks. Ξ⊂C.
R	Set of tasks with encouraged redundancy. R⊂(C∖Ξ).
***Model variables***	
$x_{s,j,t}$	Decision variable. Equals 1 when, in subsystem *s*, job *j* is running at instant *t*; else, equals 0. $x_{s,j,t}\in \left\{0,1\right\}$.
$\phi_{s,j,t}$	Auxiliary variable. Equals 1 when, in subsystem *s*, job *j* is starting its execution at instant *t*; else, equals 0. $\phi_{s,j,t}\in \left\{0,1\right\}$.
$\alpha_{s,j,t}$	Auxiliary variable. Equals 1 when, in subsystem *s*, job *j* is ending its execution at instant *t*; else, equals 0. $\alpha_{s,j,t}\in \left\{0,1\right\}$.
$\beta_{s,s^{\prime },j,t}$	Auxiliary variable. Equals 1 when, in subsystems *s* and $s^{\prime }$, job *j* is running at instant *t*; else, equals 0. $\beta_{s,s^{\prime },j,t}\in \left\{0,1\right\}$.
$b_{s,t}$	Auxiliary variable. Equals 1 when, in subsystem *s*, the battery is being charged at instant *t*; else, if the battery is being
	discharged, it equals 0. $b_{s,t}\in \left\{0,1\right\}$.
$i_{s,t}$	Battery current, in Ampere, for subsystem *s*, at instant *t*. $i_{s,t}\in R$.
$SoC_{s,t}$	State of charge (SoC) of the battery at instant *t* and subsystem *s*. $SoC_{s,t}\in R^{+},\leq 1$.

**Table 2:** Model inputs.

Notation	Description
*M*	A large number which would not be part of any optimal solution, used in big-M constraints. M∈N.
$V_{b}$	Nominal battery voltage. $V_{b}\in R^{+}$.
$SoC_{s,0}$	Initial state of charge (SoC) of the battery of subsystem *s*. $SoC_{s,0}\in R^{+},\leq 1$.
$e_{c}$	Battery charging efficiency. $e_{c}\in R^{+},\leq 1$.
$e_{d}$	Battery discharging efficiency. $e_{d}\in R^{+},\leq 1$.
*Q*	Battery nominal capacity (Ampere-hour). $Q\in R^{+}$.
ρ	Minimum accepted battery SoC. $\rho \in R^{+},<1$.
$r_{s,t}$	Power input from solar panels at subsystem *s* and instant *t*. $r_{s,t}\in R^{+}$.
$q_{s,j}$	Power required to execute job *j* in subsystem *s*. $q_{s,j}\in R^{+}$.
$u_{s,j,t}$	Dynamic priority of job *j*, at instant *t* in subsystem *s*. $u_{s,j,t}\in W$.
$t_{s,j}^{min/max}$	Minimum/maximum time required to execute job *j* in subsystem *s*. $t_{s,j}^{min/max}\in N$.
$y_{s,j}^{min/max}$	Minimum/maximum desired number of executions (startups) of job *j* in subsystem *s*. $y_{s,j}^{min/max}\in W$.
$g_{j}^{min/max}$	Minimum/maximum desired number of executions (startups) of job *j*, considering the whole constellation (all
	subsystems). $g_{j}^{min/max}\in W$.
$p_{s,j}^{min/max}$	Minimum/maximum period between executions of job *j* in subsystem *s*. $p_{s,j}^{min/max}\in N$.
$w_{s,j}^{min/max}$	Beginning(min)/ending(max) of execution window for job *j* in subsystem *s*. $w_{s,j}^{min/max}\in W$.
$\psi_{j}^{min/max}$	Minimum/maximum number of subsystems allowed to execute job *j* at the same instant (simultaneously).
	$\psi_{j}^{min/max}\in W$.

**Table 3:** Orbital parameters.

Orbit inclination [${}^{\circ }$]	97.95
Angle of the ascending node [${}^{\circ }$]	225.78
Eccentricity [-]	0.0016
Argument of perigee [${}^{\circ }$]	111.38
Mean anomaly [${}^{\circ }$]	248.91
Mean motion [rev/day]	14.82

**Table 4:** Nanosatellite tasks data (3U Mission).

*s*	Satellites 1–2 (Constellation Tasks)					Satellite 1 (Individual Tasks)					Satellite 2 (Individual Tasks)				
*j*	1	2 (Sync.-Equation (14))	3	4	5	6	7	8	9	10	6	7	8	9	10
$u_{j}$	1	10	4	8	2	3	9	6	5	7	8	2	10	9	5
$q_{j}$	2.81	0.59	1.51	2.44	2.88	0.68	1.46	2.95	1.95	2.34	2.47	2.26	1.69	1.18	2.29
$y_{j}^{\min}$	0	2	0	2	2	1	2	2	2	2	3	1	3	3	2
$y_{j}^{\max}$	9	5	4	8	8	3	9	5	4	5	5	8	5	8	6
$g_{j}^{\min}$	2	2	3	3	2	-	-	-	-	-	-	-	-	-	-
$g_{j}^{\max}$	40	23	18	37	40	-	-	-	-	-	-	-	-	-	-
$t_{j}^{\min}$	8	1	5	10	2	8	8	8	9	6	10	10	5	4	9
$t_{j}^{\max}$	19	6	21	14	3	8	17	23	21	23	18	11	21	4	14
$p_{j}^{\min}$	22	9	23	17	18	17	22	24	24	24	24	16	22	9	24
$p_{j}^{\max}$	72	30	88	59	82	83	37	79	40	80	31	27	86	71	43
$w_{j}^{\min}$	0	0	0	0	0	0	0	0	0	0	0	0	0	0	0
$w_{j}^{\max}$	96	96	96	96	96	96	96	96	96	96	96	96	96	96	96

**Table 5:** Battery parameters for each 3U nanosatellite in the constellation.

γ	ρ	*Q*	$V_{b}$	$e_{c}/e_{d}$	${\mathrm{SoC}}_{0}$
5 A	0.6	5 Ah	3.6 V	0.9	75%

## Supplementary Materials

The following supporting information can be downloaded at: https://github.com/b-rib/ETaSCoN.
